# Trends in using of antihypertensive medication among US CKD adults, NHANES 2001–2018

**DOI:** 10.3389/fcvm.2023.990997

**Published:** 2023-02-09

**Authors:** Fanghua Li, Anbang Sun, Feng Wu, Dongshan Zhang, Zhanzheng Zhao

**Affiliations:** ^1^Department of Nephrology, The First Affiliated Hospital of Zhengzhou University, Zhengzhou, Henan, China; ^2^Department of Anatomy, Tongji Medical College, Huazhong University of Science and Technology, Wuhan, China; ^3^Department of Emergency Medicine, The Second Xiangya Hospital of Central South University, Changsha, Hunan, China; ^4^Emergency Medicine and Difficult Diseases Institute, The Second Xiangya Hospital of Central South University, Changsha, Hunan, China

**Keywords:** chronic kidney disease, hypertension, blood pressure (BP), antihypertensive agent, clinical practice guidelines

## Abstract

**Objective:**

Blood pressure (BP) control rates among adult patients taking antihypertensive medications in the United States have not improved over the last decade. Many CKD adults require more than one class of antihypertensive agent to reach the BP target recommended by the guidelines. However, no study has quantified the proportion of adult CKD patients taking antihypertensive medication who are on monotherapy or combination therapy.

**Methods:**

National Health and Nutrition Examination Survey data during 2001-2018 was used, including adults with CKD taking antihypertensive medication (age ≥ 20 years, *n* = 4,453). BP control rates were investigated under the BP targets recommended by the 2021 KDIGO, the 2012 KDIGO, and the 2017 ACC/AHA guidelines.

**Results:**

The percentages of uncontrolled BP among US adults with CKD taking antihypertensive medication were 81.4% in 2001-2006 and 78.2% in 2013-2018. The proportion of monotherapy of antihypertensive regimen were 38.6, 33.3, and 34.6% from 2001 to 2006, 2007-2012, and 2013-2018, with no obvious difference. Similarly, there was no significant change in percentages of dual-therapy, triple-therapy, and quadruple-therapy. Although proportion of CKD adults not treated with ACEi/ARB decreased from 43.5% in 2001-2006 to 32.7% in 2013-2018, treatment of ACEi/ARB among patients with ACR > 300 mg/g had no significant change.

**Conclusion:**

The BP control rates among US adult CKD patients taking antihypertensive medications have not improved from 2001 to 2018. Mono-therapy accounted for about one third of adult CKD patients taking antihypertensive medication and not changed. Increasing antihypertensive medication combination therapy may help improve BP control in CKD adults in the United States.

## Introduction

The Systolic blood pressure (SBP) of < 120 mmHg reduced cardiovascular disease (CVD) risk by 25% and all-cause mortality by 27% when compared with SBP < 140 mmHg in participants with and without CKD in the Systolic Blood Pressure Intervention Trial (SPRINT) (1). Although the intensive blood pressure (BP) control group lost the estimated glomerular filtration rate (eGFR) faster and had a higher risk of chronic kidney disease (CKD) at the time, the longitudinal subgroup analysis in SPRINT discovered that the rapid loss of eGFR was reversible over the next four years (2). The 2021 Kidney Disease Improving Global Outcomes (KDIGO) guideline recommends an intensive target for blood pressure control (SBP < 120 mmHg) for all patients with CKD (3).

The proportion of US adults with hypertension taking antihypertensive medication increased to 80.5% in 2015-2018 (4, 5). However, the rate of BP control among US adults taking antihypertensive medications has been unchanged significantly over the last decade (6). The SPRINT initial therapy for the intensive BP control group was dual-therapy or triple-therapy (1). In line with this, the 2018 European Society of Cardiology-European Society of Hypertension (ESC-ESH) guideline recommended dual-therapy for initial treatment, because monotherapy is less likely to control BP under the recommended target (7).

Angiotensin-converting enzyme inhibitors (ACEi) or Angiotensin II-receptor blockers (ARB) were recommended by 2021, 2012 KDIGO guidelines and 2017 American College of Cardiology (ACC)/American Heart Association (AHA) BP guideline for CKD patients with BP above the target (3, 8, 9). The 2017 ACC/AHA BP guideline also recommends dual-therapy as initial antihypertensive medication for adults with SBP/DBP (diastolic blood pressure) ≥ 140/90 mmHg and average BP > 20/10 mmHg above their BP goals (8).

Several previous studies have evaluated trends in antihypertensive agent use among US adults with CKD (10, 11). Most of them concentrated solely on the ACEi or ARB, while information on antihypertensive medication regimens, such as monotherapy and combination therapy, and treatment adequacy in US adult CKD patients with hypertension was scarce. To this end, we used National Health and Nutrition Examination Survey (NHANES) 2001-2018 data to study the trends in antihypertensive monotherapy and combination, as well as examine associations between antihypertensive medication classes and blood pressure control in US adults with CKD and hypertension.

## Materials and methods

### Data source

NHANES is a population-based survey conducted by the National Center for Health Statistics of the US Centers for Disease Control and Prevention (CDC), and the data used in this study were downloaded from the National Center for Health Statistics (https://wwwn.cdc.gov/nchs/nhanes/default.aspx). NHANES uses a stratified, multi-stage probabilistic sampling approach to select participants. Consequently, the results can be used to estimate the health and nutrition status of the civilian, non-institutionalized population in the US. The survey in NHANES is cross-sectional and has been conducted in every two-year cycle. Therefore, we pooled data from 2001-2018, and grouped it into three separate groups: 2001-2006, 2007-2012, and 2013-2018.

### Data collection

In addition to interviews and physical examinations, NHANES data includes laboratory tests on blood and urine samples. The interviews were conducted in-home, and physical and laboratory measurements were in a mobile examination center. All the participants have provided written informed consent. A list of covariates included in this study and their assessment methods are revealed in Supplementary Table 1.

### Study population

Based on the Chronic Kidney Disease Epidemiology Collaboration equation (12), CKD was defined as estimated glomerular filtration rate (eGFR) < 60 mL/min/1.73m^2^ or as the urinary albumin-to-creatinine ratio (ACR) ≥ 30 mg/g, and the serum creatine and urinary albumin and creatinine were from NHANES single measurement. For our analytic population, we included participants who had eGFR < 60 ml/min/1.73 m^2^, or those who had eGFR ≥ 60 ml/min/1.73 m^2^ and ACR ≥ 30 mg/g (*n* = 9,805). We excluded 2,642 who were less than 20 years old or were pregnant. There were 7,163 participants have completed three times SBP and DBP measurements, and answered: “yes” to “Have you ever been told by a doctor that you had hypertension, also called high BP?” (13), and to “Are you now taking prescribed medicine for high BP?”. Those who did not have information on antihypertensive medication use during the pill container review (*n* = 2,710) were excluded. The final sample for the current analysis consisted of 4,453 participants with complete information on eGFR and three times blood pressure measurements, self-reported hypertension, and antihypertensive medication information (the population selection shown in Supplementary Figure 1).

### Blood pressure measurement

Trained study physicians performed measurements of BP according to a standardized protocol (14). Three consecutive BP measurements were taken with a mercury sphygmomanometer and an appropriate cuff size after a five-minute rest. If the BP measurement was incomplete or interrupted, the fourth BP measurement was taken. We calculated the mean SBP and DBP for each participant using three measurements.

### Antihypertensive medication use

We obtained information on antihypertensive medication use from the questionnaire including the two parts. First, answered “yes” to “Are you now taking prescribed medication for high BP?” (13). Second, participants showed the container of prescription and non-prescription medications and supplements taken in the previous 30 days (15). The antihypertensive medication was classified using the Multum Lexicon 3-level nested category system, and all reported drug names were recorded as standard generic drugs. The fixed-dose combination (FDC) products were divided into individual generic ingredients (e.g., irbesartan/hydrochlorothiazide was classified into two separate compounds, “irbesartan” and “hydrochlorothiazide”). There are four classes of antihypertensive agents: ACEi/ARB, diuretics, beta-blockers (BB), and calcium channel blockers (CCB). In the current analysis, ACEi and ARB were classified into one group, and diuretics (thiazide, loop, and potassium-sparing) as one group.

### Recommended BP targets

BP targets were recommended by the 2021 KDIGO guideline (3), the 2012 KDIGO guideline (9), and the 2017 ACC/AHA guideline (8). The 2012 KDIGO guideline recommended SBP < 120 mmHg as the BP target. The 2021 KDIGO guideline recommended SBP ≤ 130 mmHg and DBP ≤ 80 mmHg for those individuals with albuminuria (ACR ≥ 30 mg/g), SBP ≤ 140 mmHg and DBP ≤ 90 mmHg for those individuals with no albuminuria (ACR < 30 mg/g). 2017 ACC/AHA guideline recommended SBP/DBP **<** 130/80 mmHg for all CKD patients.

### Analysis

Among US adults with CKD using an antihypertensive agent, the following characteristics were analyzed: age, sex, race/ethnicity, and education in three calendar periods: 2001-2006, 2007-2012, and 2013-2018. The linear regression for continuous variables and logistic regression for binary variables were used to evaluate the trend in participants’ characteristics across calendar periods. In addition, we calculated the distribution of sociodemographic (age, sex, race/ethnicity, and education) and clinical characteristics eGFR categories (G1/G2: eGFR ≥ 60, G3: eGFR 30-59, G4: eGFR 15-29 mL/min/1.73m^2^) (11), ACR categories (ACR < 30, ACR 30-300, ACR ≥ 300 mg/g), obesity, current smoker, diabetes, history of heart failure and stroke, and Framingham risk score across each calendar period. In each calendar, we estimated the percentage of US adults with CKD using antihypertensive agents, including monotherapy and combination therapy. We evaluated the trends in the percentage of US adult CKD participants using the calendar period modeled as a continuous independent variable and the antihypertensive agent regimen as a dependent variable.

We also calculated the proportions of antihypertensive agent use among adult CKD individuals with controlled and uncontrolled BP based on the 2021 KDIGO guideline. Furthermore, according to the 2021 KDIGO, 2012 KDIGO, and 2017 ACC/AHA BP guidelines, we estimated the proportions of adults with CKD using each antihypertensive agent and combinations of classes in uncontrolled BP groups. The distribution of antihypertensive medication classes in CKD G1/2 and G3, ACR < 30 mg/g and ACR ≥ 30 mg/g, CKD with or without diabetes, age 60-74 and ≥ 75 years old, and Non-Hispanic White (NHW) and Non-Hispanic Black (NHB) were also analyzed. All analyses were conducted based on NHANES survey weights, strata, and complex sampling design units. R version 4.1.3 (R Project for Statistical Computing) was used in all analyses, and p < 0.05 were accepted as statistically significant.

## Results

### Population characteristics

As presented in Table 1, females and NHW, CKD G3, and ACR (30-300 mg/g) constitute the majority of US adults with CKD using antihypertensive agents in every calendar period. The proportions of US adult CKD patients using antihypertensive agents who were 40-74 years old, other races, a high school diploma or higher, obesity, diabetes, CKD G1/2 and ACR ≥ 30 mg/g had increased. The proportion of those who were older than 75 years old, NHW, lower than high school diploma, house income less than $44,999, CKD G3, and ACR < 30mg/g had decreased. The proportion of US adults with CKD G1/2 (eGFR ≥ 60 mL/min/1.73m^2^) taking antihypertensive medication had increased from 31.2% in 2001-2006 to 41.47% in 2013-2018, and the proportion decreased in CKD G3 (30 ≤ eGFR **<** 59 mL/min/1.73m^2^) and groups, while the proportion had not changed in CKD G4/5 from 2001-2018 (Supplementary Table 2 and Supplementary Table 3).

**TABLE 1:** Characteristics of US adults with CKD taking antihypertensive agent in NHANES 2001-2006, 2007-2012, and 2013-2018.

Characteristic	Calendar period			
	**2001-2006 (*n* = 1,112)**	**2007-2012 (*n* = 1,659)**	**2013-2018 (*n* = 1,682)**	***P***-**value**
**Age (years),%**				
<40	2.3 (1.4-3.2)	2.2 (1.5-2.9)	1.6 (1.0-2.2)	0.16
40-59	13.6 (11.6-15.6)	15.3 (13.6-17.0)	17.4 (15.6-19.2)	0.006
60-74	37.7 (34.8-40.5)	39.2 (36.9-41.6)	41.6 (39.3-44.0)	0.033
≥ 75	46.4 (43.5-49.3)	43.3 (40.9-45.7)	39.4 (37.0-41.7)	< 0.001
Female,%	51.1 (48.1-54.0)	50.8 (48.4-53.2)	49.6 (47.3-52.0)	0.43
**Race/Ethnicity,%**				
Non-Hispanic White	62.3 (59.5-65.2)	52.2 (49.8-54.6)	46.7 (44.3–49.1)	< 0.001
Non-Hispanic Black	21.1 (18.7-23.5)	24.2 (22.2-26.3)	24.1 (22.1-26.2)	0.09
Mexican American	12.5 (10.6-14.4)	10.0 (8.6-11.5)	10.8 (9.3-12.3)	0.24
Others	4.0 (2.9-5.2)	13.6 (11.9-15.2)	18.3 (16.5-20.2)	< 0.001
**Education,%**				
< High school	38.2 (35.4-41.1)	36.5 (34.2-38.8)	26.8 (24.6-28.9)	< 0.001
High school graduate and some college	47.8 (44.8-50.7)	49.4 (47.0-51.8)	54.9 (52.6-57.3)	< 0.001
College graduate	13.8 (11.7-15.8)	14.0 (12.4-15.7)	18.1 (16.3-20.0)	< 0.001
**Household income ($),%**				
< 44,999	65.3 (62.5-68.1)	66.7 (64.5-69.0)	60.0 (57.6-62.3)	< 0.001
45,000-74,999	15.4 (13.3-17.5)	16.0 (14.3-17.8)	15.7 (14.0-17.4)	0.85
≥ 75,000	12.8 (10.8-14.7)	13.0 (11.3-14.6)	18.0 (16.1-19.8)	< 0.001
Health insurance,%	95.5 (94.3-96.7)	93.2 (92.0-94.4)	95.2 (94.0-96.4)	0.27
Had usual health care facility	98.7 (98.0-99.3)	98.0 (97.3-98.7)	96.9 (96.1-97.7)	0.002
Had health care visit in past year	99.0 (98.4-99.6)	98.1 (97.5-98.8)	97.9 (97.2-98.6)	0.02
Smoker,%	54.4 (51.5-57.3)	52.6 (50.2-55.0)	51.5 (49.2-53.9)	0.14
Obesity,%	41.7 (38.8-44.6)	49.5 (47.1-51.9)	50.7 (48.3-53.0)	< 0.001
Diabetes,%	39.3 (36.4-42.2)	46.2 (43.8-48.6)	47.0 (44.6-49.4)	< 0.001
**eGFR (mL/min/1.73m^2^),%**				
≥ 60**^#^**	31.2 (28.5-33.9)	38.3 (36.0-40.7)	41.7 (39.3-44.0)	< 0.001
30-59	61.6 (58.7-64.5)	54.4 (52.0-56.8)	51.2 (48.8-53.6)	< 0.001
≤ 29	7.2 (5.7-8.7)	7.3 (6.0-8.5)	7.1 (5.9-8.4)	0.94
**ACR(mg/g),%**				
< 30**^#^**	44.0 (41.1-46.9)	36.4 (34.1-38.7)	33.9 (31.7-36.2)	< 0.001
30-300	42.6 (39.7-45.5)	49.5 (47.1-51.9)	49.3 (47.0-51.7)	0.001
>300	10.5 (8.7-12.3)	11.3 (9.8-12.9)	13.6 (11.9-15.2)	0.008
**SBP (mmHg),%**				
<120	19.6 (17.3-21.9)	23.7 (21.6-25.7)	22.3 (20.3-24.3)	0.16
120-129	17.5 (15.3-19.8)	16.5 (14.7-18.3)	17.3 (15.5-19.1)	0.95
130-139	19.2 (16.9-21.6)	19.7 (17.8-21.6)	18.8 (17.0-20.7)	0.74
≥140	43.6 (40.7-46.5)	40.1 (37.7-42.4)	41.6 (39.2-43.9)	0.38
**DBP(mmHg),%**				
<80	79.5 (77.1-81.9)	82.8 (80.9-84.6)	81.2 (79.3-83.0)	0.39
80-89	13.8 (11.8-15.9)	10.5 (9.1-12.0)	12.6 (11.0-14.2)	0.51
≥90	6.7 (5.2-8.1)	6.7 (5.5-7.9)	6.2 (5.1-7.4)	0.63
BP above 2021 KDIGO target,%	81.4 (79.1-83.7)	77.0 (75.0-79.1)	78.2 (76.2-80.2)	0.16
BP above 2012 KDIGO target,%	55.0 (52.1-58.0)	51.7 (49.3-54.1)	55.7 (53.3-58.1)	0.52
BP above 2017 ACC/AHA target,%	58.8 (56.0-61.7)	62.1 (59.8-64.4)	62.5 (60.2-64.9)	0.11

Numbers in table are expressed as column percentages (95% confidence interval). eGFR, estimated glomerular filtration rate; ACR, albumin-to-creatinine ratio; BMI, body mass index; BP, blood pressure; SBP, systolic blood pressure; DBP, diastolic blood pressure; KDIGO, kidney disease improving global outcomes; ACC, american college of cardiology; AHA, american heart association; NHANES, national health and nutrition examination survey. ^#^eGFR ≥ 60 ml/min/1.73m^2^ participants were defined as having CKD based on the presence of albuminuria; ACR < 30 mg/g participants were defined as having CKD based on the decreased eGFR.

Table 1 also showed that the percentage of adult CKD patients using antihypertensive treatment with uncontrolled BP was up to 81.4 in 2001-2006, and decreased slightly to 78.2% in 2013-2018 (P = 0.16). Following this, the proportion was 55.0% and 55.7% according to the 2012 KDIGO guideline, and 58.8% and 62.5% according to the 2017 ACC/AHA guideline.

### Antihypertensive medication use

As shown in Table 2 and Supplementary Figure 2, the percentages of adults with CKD using a single class of antihypertensive agents were 38.6, 33.3, and 34.6% from 2001-2006, 2007-2012, and 2013-2018, without significant difference. Similarly, there was no apparent change in the proportion of two, three, four or more classes of antihypertensive medication. Table 2 displays that ACEi/ARB was the most common monotherapy for hypertension and remained unchanged from 2001 to 2018, but proportion of CKD adults not treated with ACEI/ARB decreased from 43.5% in 2001-2006 to 32.7% in 2013-2018. The monotherapy of diuretics and decreased from 7.6 and 3.7% between 2001-2006 to 2013–2018.

**TABLE 2:** Proportion of antihypertensive agent use among US adults with CKD in 2001-2006, 2007-2012, and 2013-2018.

Antihypertensive regimens	Calendar period			
	**2001-2006 (*n* = 1,112)**	**2007-2012 (*n* = 1,659)**	**2013-2018 (*n* = 1,682)**	***P**-* **value**
One medication class,%	38.6 (35.7-41.4)	33.3 (31.0-35.5)	34.6 (32.3-36.9)	0.09
ACEi/ARB,%	13.8 (11.8-15.9)	15.3 (13.5-17.0)	16.3 (14.5-18.1)	0.08
Diuretic,%	7.6 (6.1-9.2)	4.8 (3.7-5.8)	3.7 (2.8-4.7)	<0.001
BB,%	7.8 (6.2-9.4)	7.7 (6.4-8.9)	8.1 (6.8-9.4)	0.77
CCB,%	7.0 (5.5-8.5)	4.5 (3.5-5.5)	5.1 (4.1-6.2)	0.06
Others,%	1.9 (1.1-2.7)	1.1 (0.6-1.6)	1.4 (0.8-1.9)	0.33
Two medication classes,%	36.8 (33.9-39.6)	33.6 (31.3-35.8)	35.9 (33.6-38.2)	0.80
ACEi/ARB + diuretic,%	12.3 (10.4-14.3)	10.8 (9.4-12.3)	10.6 (9.1-12.1)	0.17
ACEi/ARB + BB,%	4.8 (3.5-6.0)	6.3 (5.2-7.5)	8.0 (6.7-9.3)	<0.001
ACEi/ARB + CCB,%	4.9 (3.7-6.2)	4.8 (3.8-5.9)	6.0 (4.9-7.1)	0.18
CCB + diuretic,%	4.1 (3.0-5.3)	1.7 (1.1-2.4)	2.5 (1.8-3.2)	0.02
BB + diuretic,%	5.5 (4.1-6.8)	5.3 (4.2-6.4)	3.9 (3.0-4.9)	0.04
CCB + BB,%	2.7 (1.7-3.7)	2.2 (1.5-2.9)	3.3 (2.5-4.2)	0.22
Three medication classes,%	18.9 (16.6-21.2)	24.0 (21.9-26.0)	18.0 (16.2-19.9)	0.35
ACEi/ARB + diuretic + BB,%	7.3 (5.8-8.8)	9.3 (7.9-10.7)	7.7 (6.4-8.9)	0.93
ACEi/ARB + diuretic + CCB,%	4.1 (3.0-5.3)	5.0 (4.0-6.1)	4.8 (3.7-5.8)	0.51
ACEi/ARB + CCB + BB,%	1.3 (0.7-2.0)	3.3 (2.5-4.2)	3.8 (2.9-4.7)	< 0.001
CCB + BB + diuretic,%	2.3 (1.4-3.2)	2.2 (1.5-2.9)	1.8 (1.2-2.5)	0.36
Four medication classes or more,%	6.0 (4.6-7.4)	9.0 (7.7-10.4)	8.4 (7.1-9.7)	0.21
ACEi/ARB + CCB + BB + diuretic,%	3.2 (2.2-4.3)	4.1 (3.1-5.1)	4.2 (3.3-5.2)	0.21
No ACEI/ARB,%	43.5 (40.6-46.4)	33.2 (30.9-35.4)	32.7 (30.5-34.9)	<0.001

Numbers in table are expressed as column percentages (95% confidence interval). ACEi, angiotensin-converting enzyme inhibitors; ARB, angiotensin II-receptor blockers; BB, beta-blockers; CCB, calcium channel blockers.

From 2001-2006 to 2013-2018, combining a diuretic with ACEi or ARB was the most frequently dual-therapy regimen, and the proportion had no obvious change, while the combination of ACEi or ARB with BB increased from 4.8% to 7.9% (P < 0.001). The diuretic with CCB or BB had slightly decreased. The triple-therapy regimen of an ACEi/ARB combined with CCB and BB increased from 1.3% to 3.8% (P < 0.001).

The most commonly used hypertensive medication was ACEi/ARB, which increased in adult CKD G1/2 or G3, in CKD adults with ACR ≤ 300 mg/g, in CKD adults with or without diabetes, in CKD adults aged 60-74 or older than 75 years, in NHW or NHB adult CKD, from 2001-2006 through 2013-2018. The use of diuretics significantly decreased in adult CKD G3-G5, in CKD adults with ACR ≤ 300 mg/g, in CKD adults with or without diabetes, in CKD adults aged more than 75 years, and in NHW CKD adults. BB increased in CKD adults aged more than 75 years, and in NHW or NHB CKD adults (Supplementary Tables 2–6).

### Antihypertensive agent use among US adult CKD patients with uncontrolled BP

Between 2001-2006 and 2013-2018, there was no apparent difference in the proportion of monotherapy, dual-therapy, triple therapy, quadruple therapy or more antihypertensive medication combination among US adult CKD patients with uncontrolled BP based on the 2021 KDIGO (Table 3), the 2012 KDIGO (Supplementary Table 7), and the 2017 ACC/AHA guidelines (Supplementary Table 8). The monotherapy of diuretic decreased between 2001-2006 and 2013-2018. The dual-therapy of ACEi/ARB with BB or triple-therapy of ACEi/ARB with BB and CCB increased among those CKD patients with BP above the targets recommended by the 2021 KDIGO (Table 3), the 2012 KDIGO (Supplementary Table 7), and the 2017 ACC/AHA guidelines (Supplementary Table 8). The proportion of adult CKD with uncontrolled BP not taking ACEI/ARB decreased between 2001-2006 and 2013-2018.

**TABLE 3:** Proportion of antihypertensive medication use among adult CKD patients with uncontrolled blood pressure (according to 2021 KDIGO guideline) taking antihypertensive agent in 2001-2006, 2007-2012, and 2013-2018.

Antihypertensive regimens	Calendar period			
	**2001-2006 (*n* = 894)**	**2007-2012 (*n* = 1,266)**	**2013-2018 (*n* = 1,307)**	***P**-* **value**
One medication class,%	38.7 (35.5-41.9)	33.7 (31.1-36.3)	35.0 (32.5-37.6)	0.12
ACEi/ARB,%	14.2 (11.9-16.5)	14.8 (12.9-16.8)	15.8 (13.9-17.8)	0.28
Diuretic,%	7.7 (6.0-9.5)	4.5 (3.4-5.6)	3.8 (2.8-4.9)	<0.001
BB,%	7.6 (5.9-9.3)	8.1 (6.6-9.6)	8.2 (6.7-9.7)	0.63
CCB,%	7.5 (5.8-9.2)	5.0 (3.8-6.2)	5.9 (4.6-7.2)	0.18
Others,%	1.7 (0.8-2.5)	1.3 (0.7-2.0)	1.3 (0.7-1.9)	0.48
Two medication classes,%	36.5 (33.3-39.6)	34.3 (31.7-36.9)	35.9 (33.3-38.5)	0.88
ACEi/ARB + diuretic,%	11.2 (9.1-13.3)	10.3 (8.7-12.0)	9.7 (8.1-11.3)	0.27
ACEi/ARB + BB,%	4.9 (3.5-6.3)	6.9 (5.5-8.3)	7.7 (6.3-9.2)	0.01
ACEi/ARB + CCB,%	5.8 (4.3-7.4)	5.0 (3.8-6.2)	6.6 (5.2-7.9)	0.35
CCB + diuretic,%	4.0 (2.7-5.3)	2.0 (1.2-2.7)	2.6 (1.7-3.5)	0.08
BB + diuretic,%	5.8 (4.3-7.4)	5.1 (3.8-6.3)	4.1 (3.1-5.2)	0.07
CCB + BB,%	2.6 (1.5-3.6)	2.5 (1.7-3.4)	3.3 (2.3-4.3)	0.28
Three medication classes,%	18.6 (16.0-21.1)	23.1 (20.8-25.5)	20.3 (18.1-22.5)	0.50
ACEi/ARB + diuretic + BB,%	6.8 (5.2-8.5)	8.5 (7.0-10.1)	6.3 (5.0-7.6)	0.46
ACEi/ARB + diuretic + CCB,%	4.3 (2.9-5.6)	5.0 (3.8-6.2)	4.7 (3.6-5.9)	0.64
ACEi/ARB + CCB + BB,%	1.6 (0.8-2.4)	3.2 (2.2-4.1)	4.1 (3.1-5.2)	<0.001
CCB + BB + diuretic,%	2.6 (1.5-3.6)	2.1 (1.3-2.8)	1.9 (1.2-2.7)	0.31
Four medication classes or more,%	6.2 (4.6-7.7)	8.8 (7.2-10.3)	8.6 (7.1-10.2)	0.05
ACEi/ARB + CCB + BB + diuretic,%	3.4 (2.2-4.5)	4.0 (2.9-5.1)	4.2 (3.1-5.3)	0.33
No ACEI/ARB,%	43.3 (40.0-46.5)	34.6 (32.0-37.2)	34.4 (31.8-36.9)	<0.001

Numbers in table are expressed as column percentages (95% confidence interval). ACEi, angiotensin-converting enzyme inhibitors; ARB, angiotensin II-receptor blockers; BB, beta-blockers; CCB, calcium channel blockers.

### Antihypertensive agent use among US adult CKD patients with controlled and uncontrolled BP

We also compared the proportion of antihypertensive agents taken among CKD adults between BP controlled and uncontrolled groups (based on 2021 KDIGO). As presented in Table 4, there was no significant difference in monotherapy, dual-therapy, triple therapy, quadruple therapy or more antihypertensive medication combination between BP controlled and uncontrolled groups. The dual-therapy of ACEi/ARB with diuretic or triple-therapy of ACEi/ARB with diuretic and BB decreased in uncontrolled BP group (Table 4). The proportion of CKD adults not taking ACEI/ARB increased in uncontrolled BP group.

**TABLE 4:** Proportion of antihypertensive medication use among adult CKD patients with controlled or uncontrolled blood pressure (according to 2021 KDIGO guideline) currently taking antihypertensive agent in 2001-2018.

Antihypertensive regimens	Controlled BP (*n* = 986)	Uncontrolled BP (*n* = 3,467)	*P*-value
One medication class,%	33.7 (30.7-36.6)	35.4 (33.8-37.0)	0.32
ACEi/ARB,%	16.5 (14.2-18.9)	14.9 (13.8-16.1)	0.22
Diuretic,%	5.3 (3.9-6.7)	5.0 (4.3-5.8)	0.78
BB,%	7.3 (5.7-8.9)	8.0 (7.1-8.9)	0.46
CCB,%	3.1 (2.1-4.2)	6.0 (5.2-6.8)	<0.001
Others,%	1.4 (0.7-2.2)	1.4 (1.0-1.8)	0.93
Two medication classes,%	34.2 (31.2-37.1)	35.6 (34.0-37.2)	0.42
ACEi/ARB + diuretic,%	14.0 (11.8-16.2)	10.3 (9.3-11.3)	0.001
ACEi/ARB + BB,%	6.1 (4.6-7.6)	6.7 (5.9-7.5)	0.50
ACEi/ARB + CCB,%	3.5 (2.4-4.7)	5.8 (5.0-6.6)	0.006
CCB + diuretic,%	1.8 (1.0-2.7)	2.9 (2.3-3.4)	0.08
BB + diuretic,%	4.7 (3.3-6.0)	4.9 (4.2-5.6)	0.79
CCB + BB,%	2.2 (1.3-3.2)	2.9 (2.3-3.4)	0.27
Three medication classes,%	24.0 (21.4-26.7)	20.9 (19.5-22.2)	0.03
ACEi/ARB + diuretic + BB,%	11.9 (9.8-13.9)	7.2 (6.3-8.0)	<0.001
ACEi/ARB + diuretic + CCB,%	4.8 (3.4-6.1)	4.7 (4.0-5.4)	0.90
ACEI/ARB + CCB + BB,%	2.6 (1.6-3.6)	3.1 (2.5-3.7)	0.44
CCB + BB + diuretic,%	4.6 (3.3-5.9)	2.2 (1.8-2.7)	0.16
Four medication classes or more,%	8.0 (6.3-9.7)	8.0 (7.1-9.0)	0.97
ACEi/ARB + CCB + BB + diuretic,%	4.0 (2.7-5.2)	3.9 (3.3-4.6)	0.96
No ACEI/ARB,%	30.5 (27.7-33.4)	37.0 (35.4-38.6)	<0.001

Numbers in table are expressed as column percentages (95% confidence interval). ACEi, angiotensin-converting enzyme inhibitors; ARB, angiotensin II-receptor blockers; BB, beta-blockers; CCB, calcium channel blockers.

### Characteristics of resistant hypertension

Resistant hypertension is defined as above BP target despite 3 or more antihypertensive agents, including a diuretic, administered at maximally tolerated doses and appropriate dosing frequency (16). In this analysis, the percentages of resistant hypertension were 17.7%, 21.8% and 19.0% in 2001-2006, 2007-2012, and 2013-2018 respectively. As shown in Supplementary Table 9, older than 75 years, NHW, high school graduate and some college, house income less than $44,999, CKD G3, and ACR (30-300 mg/g) accounted for the majority of US adult CKD with resistant hypertension in every calendar period. The proportions of US adult CKD patients with resistant hypertension who were 40-59 years old and other races, obesity, diabetes, CKD G1/2 and ACR ≥ 30 mg/g had increased. The proportions of those who were older than 75 years old, NHW, lower than high school diploma, CKD G3, and ACR < 30 mg/g had decreased, while the proportion had not changed in CKD G4/5 from 2001-2018. As presented in Supplementary Table 10, among CKD adults taking antihypertensive medication, those aged 20-59 and 60-74 years old were less likely on resistant hypertension versus patients older than 75 years (PR: 0.69, 95% CI: 0.53-0.89, and PR: 0.77, 95% CI: 0.64-0.93, respectively). Similarly, adult CKD patients who had household income more than $75,000 were less likely on resistant hypertension vs. those who had household income less than $44,999. was more likely in those Non-Hispanic Black vs. Non-Hispanic White (PR: 1.49, CI: 1.22-1.81). Among CKD adults taking antihypertensive medication, those with diabetes, overweight, obesity, CKD G3-5, ACR ≥ 300 mg/g were more likely on resistant hypertension.

## Discussion

SPRINT revealed that the intensive SBP control (SBP < 120 mmHg) was associated with a reduction of CVD and all-cause mortality risk for patients with and without CKD (1). The longitudinal study of the CKD subgroup in SPRINT suggested that the rapid loss of eGFR was reversible (17). The SPRINT *post hoc* analysis showed that the intensive SBP control reduced CVD events and mortality independent of albuminuria (18). Another analysis verified that if intensive SBP control criteria of SPRINT were implemented, about 107,500 deaths could be prevented yearly (19). The extended follow-up (about 10 years) of the Modification of Diet in Renal Disease Study (MDRD) found that the intensive BP control could slow the progression of CKD (20). Furthermore, the long-term follow-up (about 19 years) of MDRD indicated that intensive BP control was associated with reduced post-ESRD mortality in CKD patients (21). Based on the trials above, the 2021 KDIGO guideline recommended SBP < 120 mmHg for adults with CKD and hypertension (3). However, the current analysis showed that the proportions of adult CKD taking antihypertensive medication with uncontrolled BP (according to the 2021 KDIGO guideline, or the 2012 KDIGO guideline, or the 2017 ACC/AHA guideline) were unchanged in recent 18 years. This may due to the unchanged proportion of mono-, dual-, triple-, and quadruple therapy, and about one-third of US adults with CKD took a single antihypertensive regimen class, even those with uncontrolled blood pressure. The unchanged high proportion of monotherapy may be caused by adult CKD patients cost, preference, and resistant to take more pills.

There were several gaps in the antihypertensive medication regimen between practice patterns and clinical practice guideline recommendations for adult CKD patients. For example, this analysis showed that about one-third of US adults with CKD take a single antihypertensive regimen class, even those with uncontrolled blood pressure. In addition, it has been demonstrated that monotherapy has less BP-lowering ability than dual-therapy and triple-therapy regimens (22–26). In line with this, the guideline of the 2018 ESC-ESH recommended dual-therapy for initial treatment, because monotherapy is less likely to control BP under the recommended target (9). The SPRINT used dual-therapy or triple-therapy as initial therapy for the intensive BP control group (1). The persistently high percentage of uncontrolled blood pressure among adults with CKD would increase hypertension-related risk of CVD and all-cause mortality (27, 28). Based on the evidence above, according to the current analysis, to achieve the 2021 KDIGO BP target, initiatives should be focused on increasing the combination of two or more antihypertensive medication classes.

The 2003 JNC 7 guideline recommended ACEi/ARB treatment for adults with urine ACR ≥ 300 mg/g (24). The 2012 KDIGO guideline recommended ACEi/ARB treatment for adults with ACR ≥ 30 mg/g when SBP > 130 mmHg or DBP > 80 mmHg (9), and the 2021 KDIGO guideline recommended ACEi/ARB treatment for urine ACR ≥ 30 mg/g when SBP ≥ 120 mmHg (3). A randomized clinical trial showed that when compared with amlodipine, ramipril has more effect on delaying the progression of renal disease in CKD patients with hypertension with or without proteinuria (27). The current analysis showed that the proportion of CKD adults not treated with ACEI/ARB decreased from 43.4% in 2001-2006 to 32.7% in 2013-2018, but the percentage of CKD patients with ACR > 300 mg/g taking ACEI/ARB had no significant change from 2001 to 2018. This may be caused by albuminuria undertesting, which leads to be under-recognized by clinician. A previous study in 2022 showed that only 9% of American veterans with hypertension who did not know CKD or diabetes had prior urine albumin/creatinine ratio measure (29). The proportion of ACEi/ARB treatment in CKD adults with ACR (30-300 mg/g) increased from 57.0% in 2001-2006 to 65.3% in 2007-2012, and remained stable thereafter. The stabled proportion of ACEi/ARB treatment after 2012 may accounted for the landmark clinical trial in 2010 that the Action to Control Cardiovascular Risk in Diabetes trial (ACCORD) (30), which suggested that SBP < 120 mm Hg in adult diabetes patients did not lower the risk of cardiovascular disease (CVD) mortality when compared with SBP < 140 mm Hg. The treatment of hypertension shifted to less intensity after that.

Both 2017 ACC/AHA and 2018 ESC-ESH guidelines recommended ACEi/ARB, CCB, and thiazide or thiazide-like diuretics as first-line antihypertensive agents (7, 8), but the current study revealed a sharp decline in US adults with CKD taking monotherapy of diuretics. β-blockers, also known as second-line antihypertensive agents, were not recommended for monotherapy in the 2017 ACC/AHA guideline (8), which were used more frequently than diuretics in the current analysis, and the proportion had no apparent change between 2001-2006 and 2013-2018. Efforts should be made to select an appropriate medication that avoids less effective, non-preferential, or harmful regimens, including using BB in adults with CKD without compelling indications that it will improve BP management.

In addition to identifying several trends in the use of antihypertensive medication, the present analysis also revealed changes in practice patterns following the recommendations of clinical practice guidelines and landmark clinical trials. For example, a study compared the effectiveness of dual-therapy for BP control in 2019, which showed that amlodipine plus either hydrochlorothiazide or perindopril significantly decreased SBP compared to perindopril plus hydrochlorothiazide at six months in black patients in Africa (31). In accordance with this, the current analysis showed a significant increase in US adults with CKD taking dual-therapy of ACEi/ARB and CCB combination, in addition, the regimen of ACEi/ARB plus diuretic or BB plus diuretic decreased. This may be influenced by another clinical trial which demonstrated that the amlodipine-perindopril regimen reduced more CVD risk than the atenolol-bendroflumethiazide regimen (32). The randomized controlled trial of Avoiding Cardiovascular Events through Combination Therapy in Patients Living with Systolic Hypertension (ACCOMPLISH) proved that CVD risk was decreased in dual-therapy with the benazepril-amlodipine group when compared with benazepril-hydrochlorothiazide in patients with hypertension who were at high risk for CVD (33). Although the current analysis revealed that ACEi/ARB combined with a diuretic regimen was the most commonly used combination therapy, the proportion had not changed from 2001 to 2018.

We also analyzed the resistant hypertension in US adult CKD. This analysis showed that the resistant hypertension was more likely occurred in diabetes, obesity, CKD G3-5, ACR ≥ 300 mg/g. Furthermore, the proportions of resistant hypertension in US adult CKD patients with obesity, diabetes, CKD G1/2 and ACR ≥ 30 mg/g were on the upward trend. Because resistant hypertension is an important risk factor for ESRD (34), it is necessary to take appropriate clinical management strategies to treat resistant hypertension to preserve the renal function.

The current study has several strengths. First, the NHANES participant population is representative of the US population due to the sampling design used. NHANES enrolled a large sample size and followed standardized laboratory measurement and examination procedures. Second, we used recent 20 years of data on antihypertensive medication use in adults with CKD for an in-depth analysis of antihypertensive medication regimens. Meanwhile, the current analysis has several limitations. Our analysis relied on only one measurement of serum creatinine and urinary albumin/creatinine, which may lead to misclassification of CKD, primarily when CKD is defined by albuminuria alone. Based on clinical guidelines, CKD is defined by a decrease in eGFR or the presence of proteinuria for at least three months (24). The medication dose records are not available in NHANES, so it is impossible to distinguish the intensity of antihypertensive medication, and even not to determine who took more than one medication for antihypertensive initial treatment. Third, the medications taken in the past 30 days were validated based on self-report, although they were verified by pill bottle review, and may not reflect the actual use. Complications, including heart failure and stroke are identified by self-report, which may lead to incorrect information. Finally, differences in trends of the antihypertensive medication in adults with CKD by gender, education, obesity, and other sociodemographic characteristics were not addressed in the current analysis.

## Conclusion

The BP control rates among US adult CKD patients taking antihypertensive medications have not improved from 2001 to 2018. Mono-therapy accounted for about one third of adult CKD patients taking antihypertensive medication and not changed from 2001 to 2018. The uncontrolled BP may be caused by insufficient antihypertensive medication use, and increasing the combination therapy regimens may lead to improvement of BP control, in turn reducing the risk of cardiovascular events and mortality, as well as slowing kidney disease progression among adults with CKD (35, 36).

## Data availability statement

The raw data supporting the conclusions of this article will be made available by the authors, without undue reservation.

## Ethics statement

The studies involving human participants were reviewed and approved by NCHS Ethics Review Board (NCHS is part of the Centers for Disease Control and Prevention). The patients/participants provided their written informed consent to participate in this study.

## Author contributions

FL had full access to all of the data in the study and take responsibility for the integrity of the data and the accuracy of the data analysis. DZ contributed to the concept and design. FL and AS drafted the manuscript and performed the administrative, technical, or material support. ZZ and DZ contributed to the critical revision of the manuscript for important intellectual content and performed the supervision. FL, AS, and FW performed the statistical analysis. All authors have data acquisition, analysis, or interpretation of data.
