# Supplementary material for: Trends in using of antihypertensive medication among US CKD adults, NHANES 2001–2018
Source: Front Cardiovasc Med. 2023 Feb 9;10:990997. doi: 10.3389/fcvm.2023.990997 (PMC9947777; doi:10.3389/fcvm.2023.990997)
Supplement: Supplementary file 1 [file Data_Sheet_1.docx]

Supplementary Material


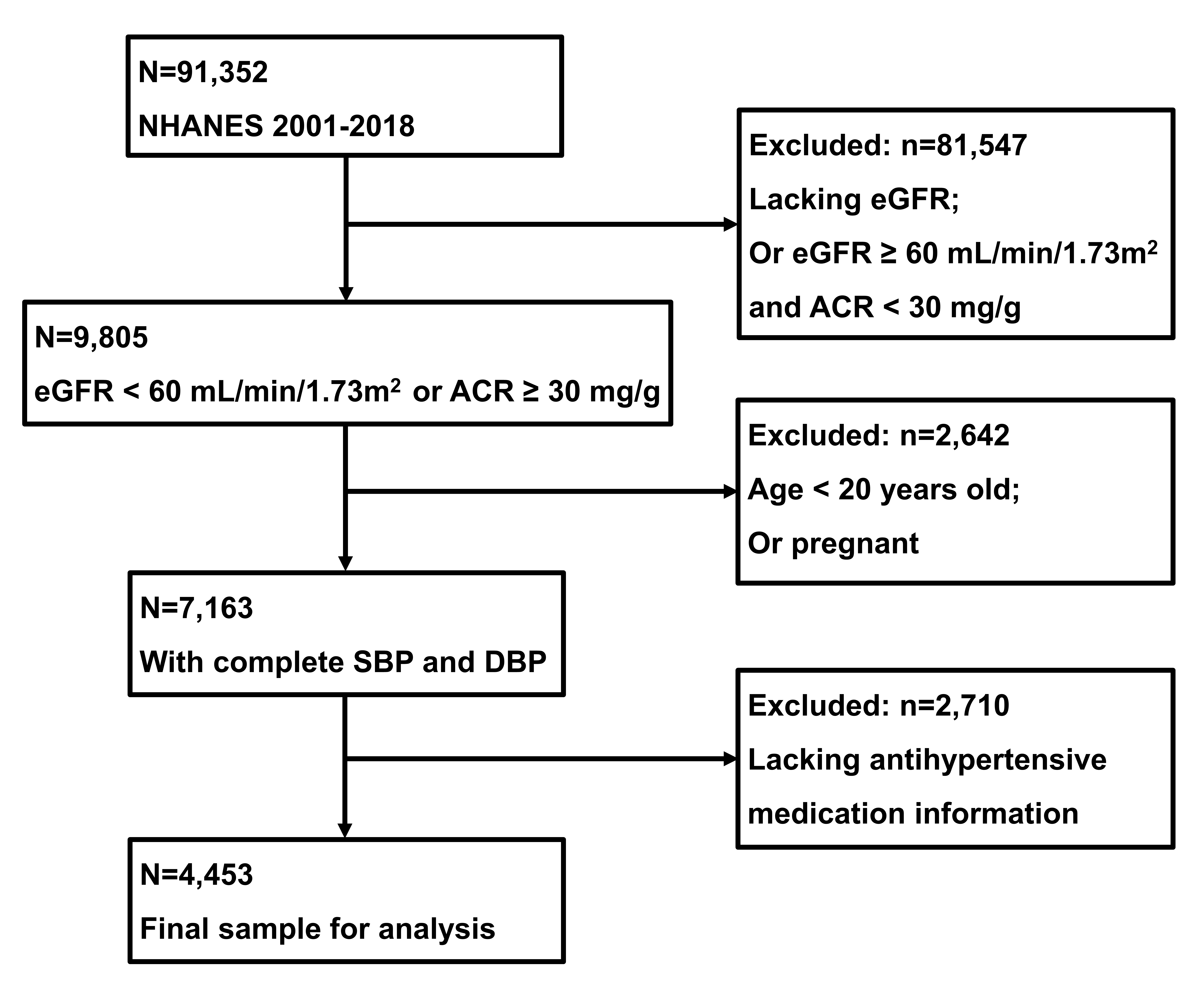


**Supplementary Figure 1.** Flowchart showing the selection of the study sample for analyzing of antihypertensive medication use in US adult CKD patients with hypertension currently taking antihypertensive agent, NHANES 2001-2018. Abbreviations: DBP, diastolic blood pressure; NHANES, National Health and Nutrition Examination Survey; SBP, systolic blood pressure.


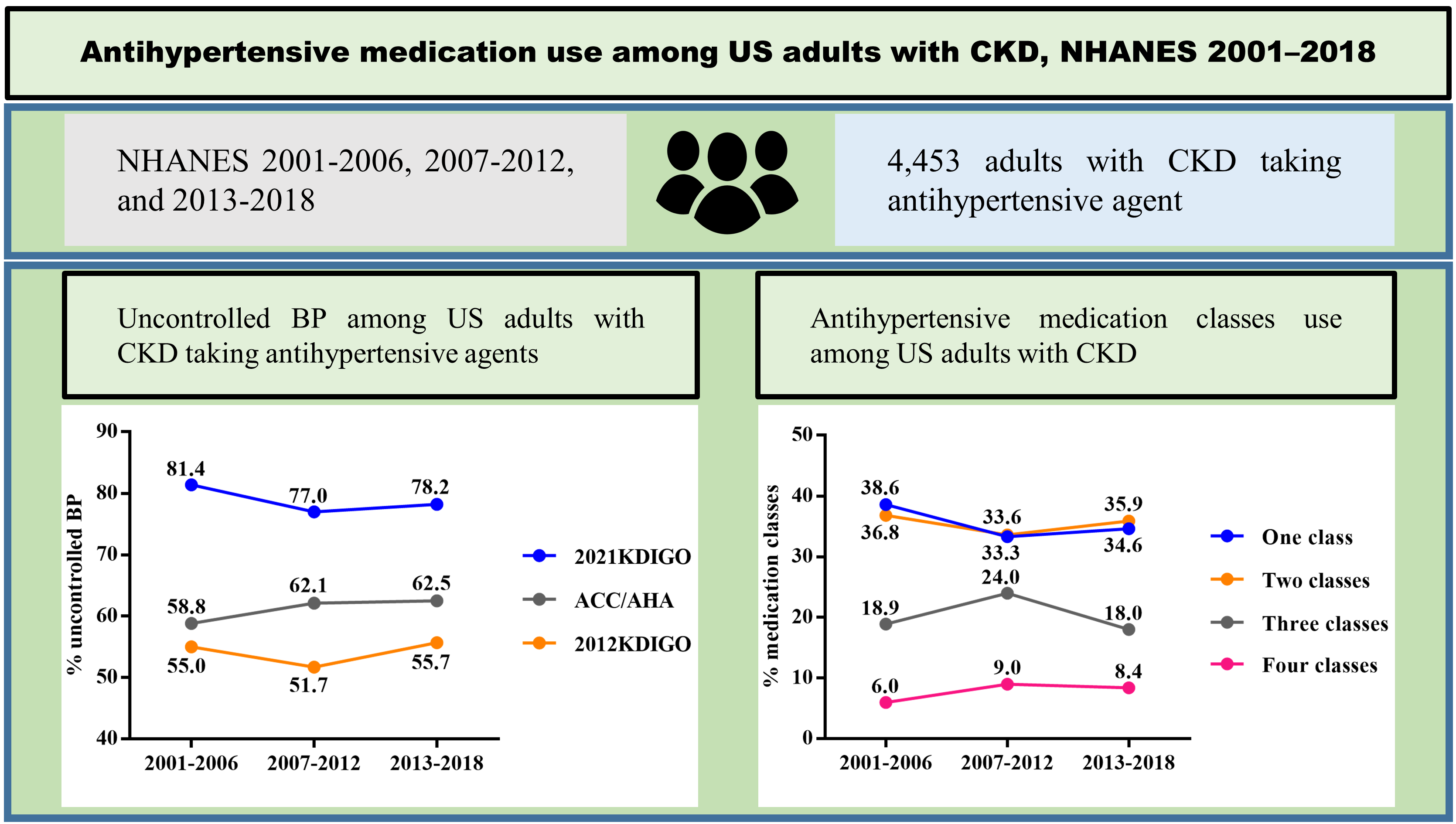


**Supplementary Figure 2.** Antihypertensive medication use among US adults with CKD, NHANES 2001–2018.

**Supplementary Table 1. Variables in the current analysis and methods of ascertainment in the NHANES, 2001-2018.**

| **Variable** | **Methods of ascertainment** |
| --- | --- |
| Age | Self-report |
| Gender | Self-report |
| Race/ethnicity | Self-report |
| Education | Self-report |
| Obesity | BMI examined at NHANES, BMI ≥30 is defined as obesity |
| Smoker | Self-report |
| Diabetes | Fasting serum glucose ≥126 mg/dL, or non-fasting serum glucose ≥200 mg/dL, or glycosylated hemoglobin  (HbA1c) ≥6.5% at NHANES examination, or self-reported use of glucose-lowing medication |
| Estimated glomerular filtration rate (eGFR) | Calculated by Chronic Kidney Disease Epidemiology Collaboration (CKD-EPI) equation based on serum creatinine measured at NHANES examination |
| Albumin-to-creatinine ratio (ACR) | Calculated by urinary albumin/urinary creatinine measured in spot urine sample measured at NHANES |
| Taking antihypertensive medication | Self-report and confirmed by pill bottle review in home |

Abbreviations: BMI, body mass index; SBP, systolic blood pressure; NHANES, National Health and Nutrition Examination Survey; eGFR, estimated glomerular filtration rate; ACR, albumin-to-creatinine ratio; BP, blood pressure.

**Supplementary Table 2. Proportion of antihypertensive medication class among adults with CKD classified by eGFR currently taking antihypertensive agent in 2001-2006, 2007-2012, 2013-2018.**

| **Medication class** | **eGFR ≥60 ml/min/1.73m^2^** | | | | **30≤ eGFR <60 ml/min/1.73m^2^** | | | | **eGFR <30 ml/min/1.73m^2^** | | | |
| --- | --- | --- | --- | --- | --- | --- | --- | --- | --- | --- | --- | --- |
|  | **2001-2006  (n=347)** | **2007-2012  (n=636)** | **2013-2020  (n=701)** | ***P* value** | **2001-2006  (n=685)** | **2007-2012  (n=902)** | **2013-2020  (n=861)** | ***P* value** | **2001-2006  (n=80)** | **2007-2012  (n=121)** | **2013-2020  (n=120)** | ***P* value** |
| Any ACEi/ARB, % | 57.1(51.9-62.3) | 67.8(64.1-71.4) | 70.8(67.4-74.1) | <0.001 | 56.8(53.1-60.5) | 67.7(64.7-70.8) | 66.2(63.0-69.4) | <0.001 | 51.3(40.3-62.2) | 55.4(46.5-64.2) | 55.0(46.1-63.9) | 0.63 |
| Any diuretic, % | 41.2(36.0-46.4) | 41.5(37.7-45.3) | 39.2(35.6-42.8) | 0.46 | 56.2(52.5-59.9) | 54.5(51.3-57.8) | 48.0(44.6-51.3) | <0.001 | 65.0(54.5-75.5) | 60.3(51.6-69.0) | 50.8(41.9-59.8) | 0.04 |
| Any BB, % | 34.3(29.3-39.3) | 41.2(37.4-45.0) | 41.2(37.6-44.9) | 0.06 | 42.3(38.6-46.0) | 50.8(47.5-54.0) | 48.1(44.7-51.4) | 0.04 | 60.0(49.3-70.7) | 57.9(49.1-66.6) | 60.8(52.1-69.6) | 0.86 |
| Any CCB, % | 34.0(29.0-39.0) | 34.4(30.7-38.1) | 37.7(34.1-41.2) | 0.19 | 30.9(27.5-34.4) | 31.0(28.0-34.1) | 33.0(29.8-36.1) | 0.37 | 47.5(36.6-58.4) | 41.3(32.5-50.1) | 46.7(37.7-55.6) | 0.98 |
| Others, % | 8.1(5.2-10.9) | 9.7(7.4-12.1) | 7.1(5.2-9.0) | 0.39 | 10.1(7.8-12.3) | 13.3(11.1-15.5) | 11.1(9.0-13.3) | 0.60 | 31.3(21.1-41.4) | 23.1(15.6-30.7) | 20.8(13.6-28.1) | 0.11 |

Numbers in table are expressed as column percentages.

Abbreviations: ACEi, angiotensin-converting enzyme inhibitors; ARB, angiotensin II-receptor blockers; BB, beta-blockers; CCB, calcium channel blockers.

**Supplementary Table 3. Proportion of antihypertensive medication class among adults with CKD classified by ACR currently taking antihypertensive agent in 2001-2006, 2007-2012, 2013-2018.**

| **Medication class** | **ACR <30 mg/g** | | | | **30≤ACR ≤300 mg/g** | | | | **ACR ＞300 mg/g** | | | |
| --- | --- | --- | --- | --- | --- | --- | --- | --- | --- | --- | --- | --- |
|  | **2001-2006  (n=489)** | **2007-2012  (n=604)** | **2013-2020  (n=571)** | ***P* value** | **2001-2006  (n=474)** | **2007-2012  (n=821)** | **2013-2020  (n=828)** | ***P* value** | **2001-2006  (n=117)** | **2007-2012  (n=188)** | **2013-2020  (n=230)** | ***P* value** |
| Any ACEi/ARB, % | 56.2(51.8-60.6) | 70.0(66.4-73.7) | 68.0(64.1-71.8) | <0.001 | 57.0(52.5-61.4) | 65.3(62.0-68.5) | 68.6(65.4-71.8) | <0.001 | 58.1(49.2-67.1) | 66.5(59.7-73.2) | 65.7(59.5-71.8) | 0.23 |
| Any diuretic, % | 57.7(53.3-62.0) | 55.8(51.8-59.8) | 49.0(44.9-53.1) | 0.004 | 47.0(42.6-51.5) | 45.2(41.8-48.6) | 41.4(38.1-44.8) | 0.04 | 50.4(41.4-59.5) | 52.1(45.0-59.3) | 43.9(37.5-50.3) | 0.15 |
| Any BB, % | 41.5(37.1-45.9) | 48.0(44.0-52.0) | 44.7(40.6-48.7) | 0.35 | 39.5(35.1-43.9) | 46.0(42.6-49.5) | 44.8(41.4-48.2) | 0.11 | 42.7(33.8-51.7) | 51.1(43.9-58.2) | 52.6(46.2-59.1) | 0.17 |
| Any CCB, % | 28.0(24.0-32.0) | 26.8(23.3-30.4) | 27.3(23.7-31.0) | 0.81 | 35.4(31.1-39.7) | 33.1(29.9-36.4) | 38.0(34.7-41.4) | 0.21 | 43.6(34.6-52.6) | 50.5(43.4-57.7) | 47.4(40.9-53.8) | 0.65 |
| Others, % | 8.6(6.1-11.1) | 11.4(8.9-14.0) | 6.8(4.8-8.9) | 0.27 | 9.9(7.2-12.6) | 11.7(9.5-13.9) | 9.4(7.4-11.4) | 0.59 | 19.7(12.5-26.9) | 22.9(16.9-28.9) | 19.6(14.4-24.7) | 0.84 |

Numbers in table are expressed as column percentages.

Abbreviations: ACEi, angiotensin-converting enzyme inhibitors; ARB, angiotensin II-receptor blockers; BB, beta-blockers; CCB, calcium channel blockers.

**Supplementary Table 4. Proportion of antihypertensive medication class among adults with CKD classified by diabetes currently taking antihypertensive agent in 2001-2006, 2007-2012, 2013-2018.**

| **Medication class** | **No diabetes** | | | | **Diabetes** | | | |
| --- | --- | --- | --- | --- | --- | --- | --- | --- |
|  | **2001-2006  (n=675)** | **2007-2012  (n=892)** | **2013-2020  (n=892)** | ***P* value** | **2001-2006  (n=437)** | **2007-2012  (n=767)** | **2013-2020  (n=790)** | ***P* value** |
| Any ACEi/ARB, % | 49.2(45.4-53.0) | 60.2(57.0-63.4) | 60.3(57.1-63.5) | <0.001 | 67.7(63.4-72.1) | 74.6(71.5-77.7) | 75.2(72.2-78.2) | 0.01 |
| Any diuretic, % | 51.3(47.5-55.0) | 47.0(43.7-50.2) | 42.3(39.0-45.5) | <0.001 | 53.5(48.9-58.2) | 53.5(49.9-57.0) | 35.2(31.9-38.5) | 0.01 |
| Any BB, % | 43.1(39.4-46.8) | 47.1(43.8-50.4) | 43.9(40.7-47.2) | 0.85 | 38.0(33.4-42.5) | 48.2(44.7-51.8) | 34.3(31.0-37.6) | 0.001 |
| Any CCB, % | 33.3(29.8-36.9) | 30.6(27.6-33.6) | 35.5(32.4-38.7) | 0.28 | 32.7(28.3-37.1) | 36.0(32.6-39.4) | 25.4(22.4-28.5) | 0.24 |
| Others, % | 11.3(8.9-13.6) | 10.4(8.4-12.4) | 8.9(7.0-10.7) | 0.11 | 10.5(7.6-13.4) | 15.3(12.7-17.8) | 7.8(6.0-9.7) | 0.94 |

Numbers in table are expressed as column percentages.

Abbreviations: ACEi, angiotensin-converting enzyme inhibitors; ARB, angiotensin II-receptor blockers; BB, beta-blockers; CCB, calcium channel blockers.

**Supplementary Table 5. Proportion of antihypertensive medication class among adults with CKD classified by age currently taking antihypertensive agent in 2001-2006, 2007-2012, 2013-2018.**

| **Medication class** | **60≤ age <75 years old** | | | | **≥75 years old** | | | |
| --- | --- | --- | --- | --- | --- | --- | --- | --- |
|  | **2001-2006  (n=419)** | **2007-2012  (n=651)** | **2013-2020  (n=700)** | ***P* value** | **2001-2006  (n=516)** | **2007-2012  (n=718)** | **2013-2020  (n=662)** | ***P* value** |
| Any ACEi/ARB, % | 59.4(54.7-64.1) | 69.9(66.4-73.4) | 70.6(67.2-73.9) | <0.001 | 52.7(48.4-57.0) | 62.3(58.7-65.8) | 61.9(58.2-65.6) | 0.002 |
| Any diuretic, % | 48.7(43.9-53.5) | 49.9(46.1-53.8) | 45.0(41.3-48.7) | 0.16 | 57.8(53.5-62.0) | 52.2(48.6-55.9) | 45.0(41.2-48.8) | <0.001 |
| Any BB, % | 42.0(37.3-46.7) | 47.2(43.3-51.0) | 44.4(40.7-48.1) | 0.58 | 41.7(37.4-45.9) | 52.9(49.3-56.6) | 50.9(47.1-54.7) | 0.003 |
| Any CCB, % | 33.4(28.9-37.9) | 34.6(30.9-38.2) | 35.4(31.9-39.0) | 0.49 | 33.1(29.1-37.2) | 33.0(29.6-36.4) | 36.9(33.2-40.5) | 0.16 |
| Others, % | 12.9(9.7-16.1) | 13.8(11.2-16.5) | 8.7(6.6-10.8) | 0.02 | 9.9(7.3-12.5) | 13.0(10.5-15.4) | 11.3(8.9-13.7) | 0.51 |

Numbers in table are expressed as column percentages.

Abbreviations: ACEi, angiotensin-converting enzyme inhibitors; ARB, angiotensin II-receptor blockers; BB, beta-blockers; CCB, calcium channel blockers.

**Supplementary Table 6. Proportion of antihypertensive medication class among NHW and NHB adults with CKD currently taking antihypertensive agent in 2001-2006, 2007-2012, 2013-2018.**

| **Medication class** | **NHW** | | | | **NHB** | | | |
| --- | --- | --- | --- | --- | --- | --- | --- | --- |
|  | **2001-2006  (n=693)** | **2007-2012  (n=866)** | **2013-2020  (n=786)** | ***P* value** | **2001-2006  (n=235)** | **2007-2012  (n=402)** | **2013-2020  (n=406)** | ***P* value** |
| Any ACEi/ARB, % | 55.0(51.3-58.7) | 64.8(61.6-68.0) | 64.8(61.4-68.1) | <0.001 | 54.9(48.5-61.3) | 66.9(62.3-71.5) | 66.0(61.4-70.6) | 0.01 |
| Any diuretic, % | 52.8(49.1-56.5) | 51.5(48.2-54.8) | 46.7(43.2-50.2) | 0.02 | 58.3(52.0-64.6) | 57.7(52.9-62.5) | 51.2(46.4-56.1) | 0.06 |
| Any BB, % | 45.0(41.3-48.7) | 52.5(49.2-55.9) | 50.6(47.1-54.1) | 0.04 | 34.0(28.0-40.1) | 43.3(38.4-48.1) | 43.6(38.8-48.4) | 0.03 |
| Any CCB, % | 30.2(26.7-33.6) | 27.1(24.2-30.1) | 31.6(28.3-34.8) | 0.51 | 44.7(38.3-51.0) | 46.0(41.1-50.9) | 47.8(42.9-52.6) | 0.44 |
| Others, % | 9.8(7.6-12.0) | 11.1(9.0-13.2) | 6.9(5.1-8.6) | 0.04 | 16.2(11.5-20.9) | 17.7(13.9-21.4) | 18.2(14.5-22.0) | 0.52 |

Numbers in table are expressed as column percentages.

Abbreviations: ACEi, angiotensin-converting enzyme inhibitors; ARB, angiotensin II-receptor blockers; BB, beta-blockers; CCB, calcium channel blockers.

**Supplementary Table 7. Proportion of antihypertensive medication use among adult CKD patients with uncontrolled blood pressure (according to 2012 KDIGO guideline) currently taking antihypertensive agent in 2001-2006, 2007-2012, 2013-2018.**

| **Antihypertensive regimens** | **Calendar period** |  |  |  |
| --- | --- | --- | --- | --- |
|  | **2001-2006  (n=612)** | **2007-2012  (n=858)** | **2013-2020  (n=937)** | ***P* value** |
| One medication class, % | 38.7(34.9-42.6) | 32.1(28.9-35.2) | 34.5(31.4-37.5) | 0.15 |
| ACEi/ARB, % | 13.9(11.1-16.6) | 13.5(11.2-15.8) | 14.9(12.7-17.2) | 0.51 |
| Diuretic, % | 6.2(4.3-8.1) | 3.0(1.9-4.2) | 3.4(2.3-4.6) | 0.01 |
| BB, % | 8.0(5.9-10.2) | 7.9(6.1-9.7) | 8.5(6.7-10.3) | 0.51 |
| CCB, % | 9.0(6.7-11.3) | 6.1(4.5-7.7) | 6.4(4.8-8.0) | 0.08 |
| Others, % | 1.6(0.6-2.6) | 1.5(0.7-2.3) | 1.2(0.5-1.9) | 0.43 |
| Two medication classes, % | 35.9(32.1-39.7) | 35.2(32.0-38.4) | 35.0(32.0-38.1) | 0.72 |
| ACEi/ARB+diuretic, % | 10.8(8.3-13.2) | 9.1(7.2-11.0) | 8.6(6.8-10.4) | 0.17 |
| ACEi/ARB+ BB, % | 5.2(3.5-7.0) | 6.8(5.1-8.4) | 8.3(6.6-10.1) | 0.02 |
| ACEi/ARB+ CCB, % | 6.7(4.7-8.7) | 5.6(4.1-7.1) | 7.0(5.4-8.7) | 0.67 |
| CCB+diuretic, % | 3.6(2.1-5.1) | 2.6(1.5-3.6) | 2.7(1.6-3.7) | 0.33 |
| BB+diuretic, % | 4.9(3.2-6.6) | 5.5(4.0-7.0) | 2.9(1.8-4.0) | 0.03 |
| CCB+BB, % | 2.5(1.2-3.7) | 2.9(1.8-4.0) | 3.3(2.2-4.5) | 0.33 |
| Three medication classes, % | 17.6(14.6-20.7) | 22.7(19.9-25.5) | 20.8(18.2-23.4) | 0.21 |
| ACEi/ARB+diuretic+BB, % | 6.7(4.7-8.7) | 8.0(6.2-9.9) | 5.7(4.2-7.1) | 0.31 |
| ACEi/ARB+diuretic+CCB, % | 3.8(2.3-5.3) | 4.3(3.0-5.7) | 5.4(4.0-6.9) | 0.11 |
| ACEi/ARB+CCB+BB, % | 1.6(0.6-2.6) | 3.4(2.2-4.6) | 4.3(3.0-5.6) | 0.006 |
| CCB+BB+diuretic, % | 2.3(1.1-3.5) | 2.4(1.4-3.5) | 1.9(1.0-2.8) | 0.58 |
| Four medication classes or more, % | 7.7(5.6-9.8) | 9.9(7.9-11.9) | 9.5(7.6-11.4) | 0.28 |
| ACEi/ARB+CCB+BB+diuretic, % | 4.1(2.5-5.7) | 4.7(3.3-6.1) | 4.6(3.2-5.9) | 0.67 |
| No ACEI/ARB, % | 41.5(37.6-45.4) | 36.2(33.0-39.5) | 33.7(30.7-36.8) | 0.02 |

Numbers in table are expressed as column percentages.

Abbreviations: ACEi, angiotensin-converting enzyme inhibitors; ARB, angiotensin II-receptor blockers; BB, beta-blockers; CCB, calcium channel blockers.

**Supplementary Table 8. Proportion of antihypertensive medication use among adult CKD patients with uncontrolled blood pressure (according to 2017 ACC/AHA guideline) currently taking antihypertensive medication in 2001-2006, 2007-2012, 2013-2018.**

| **Antihypertensive regimens** | **Calendar period** | | | |
| --- | --- | --- | --- | --- |
|  | **2001-2006  (n=732)** | **2007-2012  (n=1030)** | **2013-2020  (n=1052)** | ***P* value** |
| One medication class, % | 39.3(35.8-42.9) | 32.3(29.5-35.2) | 35.6(32.8-38.5) | 0.19 |
| ACEi/ARB, % | 13.8(11.3-16.3) | 13.9(11.8-16.0) | 15.7(13.5-17.9) | 0.24 |
| Diuretic, % | 7.4(5.5-9.3) | 3.2(2.1-4.3) | 4.0(2.8-5.2) | 0.002 |
| BB, % | 8.2(6.2-10.2) | 8.4(6.7-10.1) | 8.4(6.7-10.0) | 0.91 |
| CCB, % | 8.5(6.5-10.5) | 5.5(4.1-6.9) | 6.4(4.9-7.8) | 0.12 |
| Others, % | 1.5(0.6-2.4) | 1.3(0.6-1.9) | 1.2(0.6-1.9) | 0.64 |
| Two medication classes, % | 35.5(32.1-39.0) | 34.3(31.4-37.2) | 35.4(32.5-38.3) | 0.98 |
| ACEi/ARB+diuretic, % | 10.8(8.5-13.0) | 10.0(8.2-11.8) | 9.1(7.4-10.9) | 0.24 |
| ACEi/ARB+ BB, % | 5.1(3.5-6.6) | 6.7(5.2-8.2) | 8.0(6.3-9.6) | 0.02 |
| ACEi/ARB+ CCB, % | 6.0(4.3-7.7) | 5.0(3.7-6.4) | 7.1(5.6-8.7) | 0.25 |
| CCB+diuretic, % | 4.0(2.5-5.4) | 2.2(1.3-3.1) | 2.6(1.6-3.5) | 0.11 |
| BB+diuretic, % | 5.3(3.7-7.0) | 5.0(3.7-6.4) | 3.3(2.2-4.4) | 0.03 |
| CCB+BB, % | 2.0(1.0-3.1) | 2.7(1.7-3.7) | 3.1(2.1-4.2) | 0.16 |
| Three medication classes, % | 18.3(15.5-21.1) | 2.3(1.4-3.3) | 19.9(17.5-22.3) | 0.23 |
| ACEi/ARB+diuretic+BB, % | 6.4(4.6-8.2) | 8.5(6.8-10.3) | 5.3(4.0-6.7) | 0.17 |
| ACEi/ARB+diuretic+CCB, % | 4.2(2.8-5.7) | 5.1(3.8-6.5) | 5.1(3.8-6.5) | 0.42 |
| ACEI/ARB+CCB+BB, % | 1.6(0.7-2.6) | 3.2(2.1-4.3) | 4.1(2.9-5.3) | 0.004 |
| CCB+BB+diuretic, % | 2.7(1.6-3.9) | 2.2(1.3-3.1) | 1.9(1.1-2.7) | 0.25 |
| four medication classes or more, % | 6.8(5.0-8.7) | 9.6(7.8-11.4) | 8.9(7.2-10.7) | 0.16 |
| ACEi/ARB+CCB+BB+diuretic, % | 3.8(2.4-5.2) | 4.5(3.2-5.7) | 4.4(3.1-5.6) | 0.61 |
| No ACEI/ARB, % | 43.0(39.4-46.6) | 35.0(32.1-38.0) | 34.3(31.4-37.2) | <0.001 |

Numbers in table are expressed as column percentages.

Abbreviations: ACEi, angiotensin-converting enzyme inhibitors; ARB, angiotensin II-receptor blockers; BB, beta-blockers; CCB, calcium channel blockers.

**Supplementary Table 9. Characteristics of CKD adults with resistant hypertension in NHANES 2001-2006, 2007-2012, 2013-2018.**

| **Characteristic** | **Calendar period** | | | |
| --- | --- | --- | --- | --- |
|  | **2001-2006 (n=197)** | **2007-2012 (n=362)** | **2013-2018 (n=319)** | ***P* value** |
| Age (years), % |  | | | |
| <40 | 2.0(0.1-4.0) | 1.4(0.2-2.6) | 0.6(0.2-1.5) | 0.16 |
| 40-59 | 9.6(5.5-13.8) | 14.6(11.0-18.3) | 17.9(13.7-22.1) | 0.01 |
| 60-74 | 38.1(31.3-44.9) | 39.2(34.2-44.3) | 40.4(35.1-45.8) | 0.59 |
| ≥75 | 50.3(43.3-57.2) | 44.8(39.6-49.9) | 41.1(35.7-46.5) | 0.04 |
| Female, % | 48.7(41.8-55.7) | 47.2(42.1-52.4) | 53.3(47.8-58.8) | 0.24 |
| Race/ethnicity, % |  | | | |
| Non-Hispanic White | 58.9(52.0-65.8) | 49.2(44.0-54.3) | 42.3(36.9-47.7) | <0.001 |
| Non-Hispanic Black | 25.9(19.8-32.0) | 31.8(27.0-36.6) | 32.6(27.5-37.7) | 0.13 |
| Mexican American | 8.1(6.7-10.5) | 6.6(4.1-9.2) | 5.0(2.6-7.4) | 0.4 |
| Others | 7.1(3.5-10.7) | 12.4(9.0-15.8) | 20.1(14.6-25.6) | 0.007 |
| Education % |  | | | |
| < high school | 35.5(28.8-42.2) | 36.7(31.8-41.7) | 29.8(24.8-34.8) | <0.001 |
| High school graduate and some college | 48.2(41.2-55.2) | 52.2(47.1-57.4) | 52.4(46.9-57.8) | 0.40 |
| College graduate | 16.2(11.1-21.4) | 11.0(7.8-14.3) | 17.6(13.4-21.7) | 0.42 |
| Household income ($), % |  | | | |
| <44999 | 67.0(60.4-73.6) | 69.9(65.2-74.6) | 63.3(58.0-68.6) | 0.29 |
| 45000-74999 | 13.7(8.9-18.5) | 13.8(10.3-17.4) | 15.4(11.4-19.3) | 0.54 |
| ≥75000 | 12.7(8.0-17.3) | 11.3(8.1-14.6) | 14.4(10.6-18.3) | 0.44 |
| Health insurance, % |  | | | |
| Had usual health care facility | 99.0(97.6-100.4) | 98.1(96.6-99.5) | 98.4(97.1-99.8) | 0.89 |
| Had health care visit in past year | 99.5(98.5-100.5) | 99.2(98.2-100.1) | 99.6(98.6-100.6) | 0.92 |
| Smoker, % | 55.3(48.4-62.3) | 54.7(49.6-59.8) | 52.0(46.6-57.5) | 0.17 |
| Obesity, % | 47.2(40.2-54.2) | 61.0(56.0-66.1) | 58.0(52.6-63.4) | <0.001 |
| Diabetes, % | 45.7(38.7-52.6) | 58.8(53.8-63.9) | 60.8(55.5-66.2) | <0.001 |
| eGFR (mL/min/1.73m^2^), % |  | | | |
| ≥60**^#^** | 21.3(15.6-27.0) | 33.4(28.6-38.3) | 40.1(34.7-45.5) | <0.001 |
| 30-59 | 67.0(60.4-73.6) | 58.0(52.9-63.1) | 49.8(44.4-55.3) | <0.001 |
| ≤29 | 11.7(7.2-16.2) | 8.6(5.7-11.4) | 10.0(6.7-13.3) | 0.66 |
| ACR (mg/g), % |  | | | |
| <30**^#^** | 41.1(34.2-48.0) | 37.3(32.3-42.3) | 24.5(19.7-29.2) | <0.001 |
| 30-300 | 41.1(34.2-48.0) | 42.3(37.2-47.4) | 51.1(45.6-56.6) | 0.01 |
| ＞300 | 14.7(9.8-19.7) | 17.7(13.7-21.6) | 21.3(16.8-25.8) | 0.05 |

Numbers in table are expressed as column percentages.

Abbreviations: eGFR, estimated glomerular filtration rate; ACR, albumin-to-creatinine ratio; BMI, body mass index; BP, blood pressure; SBP, systolic blood pressure; DBP, diastolic blood pressure; KDIGO, Kidney Disease Improving Global Outcomes; ACC, American College of Cardiology; AHA, American Heart Association; NHANES, National Health and Nutrition Examination Survey.

^#^eGFR ≥60 mL/min/1.73m^2^ participants were defined as having CKD based on the presence of albuminuria; ACR <30 mg/g participants were defined as having CKD based on the decreased eGFR.

**Supplementary Table 10. Factors associated with resistant hypertension among adult CKD with hypertension in NHANES 2001-2018.**

| **Characteristic** | **Adult CKD with hypertension (n=4,453)** | | |
| --- | --- | --- | --- |
|  | **Prevalence ratio %, (95% CI)** | | |
|  | Model 1 | Model 2 | Model 3 |
| Age (years), % |  | | |
| 20-59 | **0.75(0.61-0.94)** | **0.71(0.55-0.90)** | **0.69(0.53-0.89)** |
| 60-74 | **0.85(0.72-1.00)** | **0.80(0.67-0.96)** | **0.77(0.64-0.93)** |
| ≥75 | 1(ref) | 1(ref) | 1(ref) |
| Sex, % |  | | |
| Female | 1(ref) | 1(ref) | 1(ref) |
| Male | 0.95(0.82-1.10) | 0.91(0.78-1.06) | 0.95(0.80-1.12) |
| Race/ethnicity, % |  | | |
| Non-Hispanic White | 1(ref) | 1(ref) | 1(ref) |
| Non-Hispanic Black | **1.63(1.36-1.95)** | **1.60(1.33-1.93)** | **1.49(1.22 -1.81)** |
| Mexican | 0.76(0.58-1.00) | 0.77(0.58-1.04) | 0.68(0.50-0.92) |
| other | 1.08(0.85-1.36) | 1.02(0.79-1.30) | 0.94(0.72-1.22) |
| Education, % |  | | |
| <High school | 1.02(0.86-1.20) | 0.95(0.80-1.13) | 0.92(0.76-1.10) |
| High school graduate and some college | 1(ref) | 1(ref) | 1(ref) |
| College graduate | 0.94(0.75-1.16) | 1.01(0.80-1.27) | 1.12(0.88-1.42) |
| Household income ($), % |  | | |
| ≤44999 | 1(ref) | 1(ref) | 1(ref) |
| 45000-74999 | 0.82(0.66-1.01) | 0.82(0.66-1.02) | 0.82(0.66-1.03) |
| ≥75000 | **0.79(0.63-0.98)** | **0.77(0.60-0.97)** | **0.78(0.61-1.00)** |
| Health insurance, % |  | | |
| insurance | 0.84(0.59-1.19) | 0.84(0.58-1.20) | 0.88(0.59-1.26) |
| None | 1(ref) | 1(ref) | 1(ref) |
| Healthcare facility, % |  | | |
| No | 1(ref) | 1(ref) | 1(ref) |
| Yes | 1.46(0.85-2.71) | 1.90(0.99-4.12) | 1.71(0.88-3.74) |
| Healthcare visit in past year, % |  |  |  |
| No | 1(ref) | 1(ref) | 1(ref) |
| Yes | **4.98(1.84-20.44)** | **6.50(2.01 -39.84)** | **5.63(1.72-34.66)** |
| Diabetes, % |  | | |
| No | 1(ref) | 1(ref) | 1(ref) |
| Yes | **1.88(1.61-2.18)** | **1.84(1.57 -2.15)** | **1.59(1.34 -1.88)** |
| BMI, % |  | | |
| Normal | 1(ref) | 1(ref) | 1(ref) |
| Overweight | **1.38(1.09-1.76)** | **1.37(1.07-1.76)** | **1.36(1.05-1.76)** |
| Obese | **2.01(1.61-2.54)** | **1.98(1.57-2.52)** | **1.86(1.45 -2.39)** |
| Smoking, % |  | | |
| Never smoked | 1(ref) | 1(ref) | 1(ref) |
| smoker | 1.05(0.91-1.23) | 1.01(0.86-1.18) | 1.03(0.87-1.21) |
| eGFR (mL/min/1.73m^2^), % |  | | |
| ≥60 | 1(ref) | 1(ref) | 1(ref) |
| 30-59 | **1.23(1.03-1.46)** | **1.19(1.00-1.42)** | **1.54(1.23-1.93)** |
| ≤29 | **1.62(1.22-2.14)** | **1.56(1.16-2.07)** | **1.53(1.08-2.13)** |
| ACR (mg/g), % |  | | |
| <30 | 1(ref) | 1(ref) | 1(ref) |
| 30-299 | 1.13(0.95-1.34) | 1.15(0.97-1.37) | 1.44(1.15-1.79） |
| ≥300 | **2.13(1.69-2.69)** | **2.25(1.77-2.86)** | **2.40(1.83-3.15）** |

Numbers in table are expressed as prevalence ratio (95% confidence interval).

Abbreviations: CKD, chronic kidney disease; CI: confidence interval; eGFR, estimated glomerular filtration rate; ACR, albumin-to-creatinine ratio; BMI, body mass index; BP, blood pressure; KDIGO, Kidney Disease Improving Global Outcomes.

eGFR ≥60 mL/min/1.73m^2^ participants were defined as having CKD based on the presence of albuminuria; ACR <30 mg/g participants were defined as having CKD based on the decreased eGFR.

Model 1: adjusted for age, sex, and race/ethnicity.

Model 2: Model 1+ education, income, health insurance, healthcare facility and healthcare visit.

Model 3: Adjusted for all characteristics listed.
